# Emerging Devices Based on Two-Dimensional Monolayer Materials for Energy Harvesting

**DOI:** 10.34133/2019/7367828

**Published:** 2019-11-09

**Authors:** Feng Ru Fan, Wenzhuo Wu

**Affiliations:** ^1^School of Industrial Engineering, Purdue University, West Lafayette, Indiana 47907, USA; ^2^Flex Laboratory, Purdue University, West Lafayette, Indiana 47907, USA; ^3^Birck Nanotechnology Center, Purdue University, West Lafayette, Indiana 47907, USA

## Abstract

Two-dimensional (2-D) materials of atomic thickness have attracted considerable interest due to their excellent electrical, optoelectronic, mechanical, and thermal properties, which make them attractive for electronic devices, sensors, and energy systems. Scavenging the otherwise wasted energy from the ambient environment into electrical power holds promise to address the emerging energy needs, in particular for the portable and wearable devices. The versatile properties of 2-D materials together with their atomically thin body create diverse possibilities for the conversion of ambient energy. The present review focuses on the recent key advances in emerging energy-harvesting devices based on monolayer 2-D materials through various mechanisms such as photovoltaic, thermoelectric, piezoelectric, triboelectric, and hydrovoltaic devices, as well as progress for harvesting the osmotic pressure and Wi-Fi wireless energy. The representative achievements regarding the monolayer heterostructures and hybrid devices are also discussed. Finally, we provide a discussion of the challenges and opportunities for 2-D monolayer material-based energy-harvesting devices in the development of self-powered electronics and wearable technologies.

## 1. Introduction

With the rapid development of portable devices, wearable electronics, and the Internet of Things, significant efforts have been devoted to developing sustainable, mobile, and distributed power sources for the energy of a new era [1–4]. Energy harvesting can address these emerging energy needs by converting ambient energy into electrical power as sustained, self-sufficient power sources, which constitute the burgeoning field of nanoenergy [5, 6]. There are many forms of energy sources that can be collected and utilized from the surrounding environment, such as mechanical energy, thermal energy, solar energy, and chemical energy. In recent years, with the advancement of nanotechnology and nanoscale materials, various effective strategies toward harvesting energy have been demonstrated. For solar and thermal energy harvesting, the application of new materials and principles has dramatically improved the performance of the devices [7–12]. For mechanical energy harvesting with nanomaterials, rapid advances have been achieved since the report of the first piezoelectric nanogenerator and the first triboelectric nanogenerator [13, 14] was demonstrated in 2006 and 2012 [2, 15, 16]. In addition, there are new techniques that can be used to collect other forms of energy.

The advancement and application of new materials have promoted the development of energy harvesting. Among these materials, atomically thin 2-D materials exhibit a wide range of unique electrical [17–19], optical [20], mechanical [21], and thermal properties, which make them promising candidates for use in the exploration of new energy-harvesting techniques [22–24]. Due to its high conductivity and transparency, monolayer graphene can be used in various photovoltaic devices and piezo-/triboelectric nanogenerators as transparent electrodes [25, 26]. Furthermore, all the *π*-bonds are exposed on the surface where they can interact with external species and physical fields, making graphene a suitable material for the hydrovoltaic effect [27, 28]. Other monolayer 2-D materials, such as the transition metal dichalcogenides (TMDs) and black phosphorus, display semiconducting behavior with band gaps in the visible spectrum and are attractive for being used as the interlayers or active layers in photovoltaic devices [18, 29, 30]. Monolayer 2-D materials that exhibit nonzero electronic band gaps and noncentrosymmetry in their monolayer crystal show a piezoelectric effect and can be used in the piezoelectric nanogenerators.

In this review, we summarize and review the key achievements in this fast-developing field of using 2-D materials for energy harvesting, focusing particularly on the device structure and output performance of monolayer material-based energy harvesters. A series of emerging energy-harvesting devices will be delineated, including the photovoltaic cells for solar energy, thermoelectric devices for thermal energy, the piezoelectric and triboelectric nanogenerators for mechanical energy, hydrovoltaic devices for water kinetic energy, and two unique techniques, monolayer MoS_2_ nanopores for the osmotic power and monolayer MoS_2_-enabled flexible rectenna for Wi-Fi-band wireless energy harvesting. Considering that monolayer 2-D materials can be integrated with various other nanomaterials to create functional devices, we also discuss the recent achievements in the monolayer heterostructures with optimized performance and hybrid nanogenerators capable of harvesting multiple types of energy. Finally, we provide our perspectives and discuss opportunities for future development and applications of the aforementioned energy-harvesting technologies.

## 2. Monolayer 2-D Materials for Energy Harvesting

There is a variety of sources available for the nanogenerators to harvest from the ambient environment, including but not limited to the energy in natural forms, such as wind, water flow, ocean waves, and solar power; mechanical energy, such as vibrations from machines and human motions; thermal energy, such as waste heat from heaters and joule heating of electronic devices; light energy from both indoor/city lighting and outdoor sunlight; and electromagnetic radiation from mobile electronic devices as well as from Wi-Fi systems. In this section, we will review the recent advances in various energy-harvesting techniques, their device structure, output performance, and future application prospects.

### 2.1. Photovoltaics and Solar Cells

Solar energy is by far the most abundant exploitable renewable energy resource. Photovoltaic technology that converts sunlight directly into electricity is becoming increasingly important in the world's renewable energy mix. Ultrathin or monolayer 2-D nanomaterials, such as graphene and TMDs, show great application potential for solar cells due to their intriguing electronic and optical properties.

**Figure 1:**
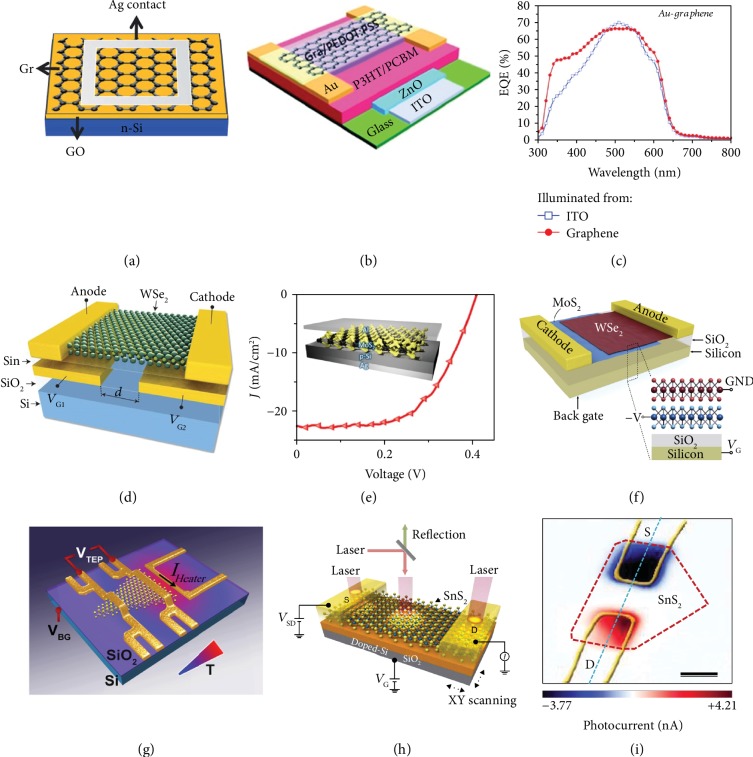
Photovoltaic and thermoelectric devices based on monolayer 2-D materials. (a) Graphene/Si Schottky junction solar cells with a GO interlayer. Reproduced with permission from the Royal Society of Chemistry [36]. (b, c) Monolayer graphene as top electrodes in semitransparent organic solar cells based on P3HT:PCBM. Reproduced with permission from the American Chemical Society [37]. (d) A p-n junction solar cell based on an electrostatically doped WSe_2_ monolayer. Reproduced with permission from the Nature Publishing Group [39]. (e) Photovoltaic devices with a monolayer MoS_2_ on p-type silicon substrates. Reproduced with permission from the American Chemical Society [41]. (f) Stacking van der Waals heterojunction using monolayers MoS_2_ and WSe_2_. Reproduced with permission from the American Chemical Society [42]. (g) Thermopower measurements of monolayer CVD-grown MoS_2_. Reproduced with permission from the American Chemical Society [54]. (h, i) Thermoelectric nanogenerator based on monolayer SnS_2_ and its laser scanning photo-induced thermoelectric current imaging. Reproduced with permission from the Nature Publishing Group [58].

With excellent electrical conductivity, high carrier mobility, and moderately high optical transmittance in the visible range of the spectrum, graphene can perform different functions in inorganic and organic solar cells, such as transparent conductive electrodes and counter electrodes [31, 32]. Compared with the traditional indium tin oxide (ITO) or fluorine-doped tin oxide (FTO) electrodes, graphene is much more flexible and therefore is more desirable for flexible solar cells [33, 34]. The monolayer graphene has an optical transparency of ~97.7% over the entire solar spectrum, with the addition of each layer corresponding to a ~2.3% decrease in transparency. Bae and coworkers reported the roll-to-roll production and wet chemical doping of predominantly monolayer 30-inch graphene films grown by chemical vapor deposition onto flexible copper substrates [35]. The films have sheet resistances as low as ∼125 *Ω*/sq with 97.4% optical transmittance, which is superior to commercial transparent electrodes such as ITO. Chemical vapor deposition (CVD) has been widely used as a bottom-up approach to produce large-area monolayer graphene transparent electrodes, which is compatible with the roll-to-roll fabrication process. CVD-grown graphene films have been used as the conducting anodes for dye-sensitized solar cells (DSSCs) and organic solar cells (OSCs), the transparent cathodes for hybrid solar cells, and catalytic counter electrodes for DSSCs. Yang et al. demonstrated a new structure of graphene/Si Schottky junction solar cells by introducing a graphene oxide (GO) interlayer to engineer the graphene/Si interface (Figure 1(a)) [36]. An optimized power conversion efficiency (PCE) of 6.18% was achieved for the graphene/GO/Si solar cells with undoped monolayer graphene as the top electrode. Liu et al. reported the use of monolayer graphene as the top electrode in semitransparent OSCs based on P3HT:PCBM, as shown in Figure 1(b) [37]. The resistance of monolayer graphene decreased to less than 100 *Ω*/sq with a transmittance of ∼90% after doping with Au nanoparticles and PEDOT:PSS. Notably, all of the devices showed higher efficiency (up to 2.7%) from the graphene than ITO side, which was attributed to the better transmittance of the graphene electrodes (Figure 1(c)). It is expected that scalably manufactured graphene would eventually replace conventional transparent oxides as the window electrode for better performance solar cells with mechanical flexibility.

Other 2-D layered materials with a band gap in the visible region of the electromagnetic spectrum can be used as photosensitizers in photovoltaics and photodetectors. Bernardi et al. reported that monolayer 2-D TMDs, e.g., MoS_2_, MoSe_2_, and WS_2_, can absorb up to 5–10% incident sunlight within a thickness of less than 1 nm, thus achieving 1 order of magnitude higher sunlight absorption than GaAs and Si [38]. Accordingly, monolayers MoS_2_ and WS_2_ have been successfully used in Schottky junction solar cells as the photoactive layers. It has been demonstrated that combining monolayer TMDs with a lateral p-n junction architecture by electrostatic doping could lead to many exciting functions including the photovoltaic effect and light-emitting properties in these atomically thin structures. For example, Pospischil et al. reported a p-n junction diode based on an electrostatically doped WSe_2_ monolayer [39]. As schematically illustrated in Figure 1(d), split-gate electrodes are coupled to two different regions of monolayer WSe_2_ flake for electrostatic doping. As a photovoltaic solar cell, a maximum electrical output power of 9 pW is obtained with a voltage of 0.64 V, a current of 14 pA, and a PCE of ~0.5%. The ∼95% transparency of the WSe_2_ monolayer makes it attractive for semitransparent solar cells. Meanwhile, a monolayer WSe_2_ device with a similar structure was prepared by Baugher et al., of which photovoltaic power generation with a peak EQE of 0.2% was demonstrated [40]. Tsai et al. reported photovoltaic devices with a monolayer MoS_2_ on p-type silicon (p-Si) substrates [41]. A built-in electric field was established near the interface between the MoS_2_ and p-Si for promoting the separation of photogenerated carriers. The current density-voltage measurement revealed a photocurrent density of 22.36 mA cm^−2^ and an efficiency of 5.23% (Figure 1(e)). This work shows that monolayer MoS_2_ could be potentially integrated into the Si manufacturing process, which holds promise for applying 2-D materials into a variety of Si-based electronic and optical devices.

It is also feasible to construct layer-stacked heterostructures with judiciously chosen monolayer materials for achieving enhanced efficiency of the photovoltaic devices. These layered designer materials are held together by van der Waals forces and contain atomically sharp interfaces. Furchi et al. reported a van der Waals heterojunction using monolayers MoS_2_ and WSe_2_ (Figure 1(f)) [42]. Upon optical illumination, charge transfer occurs across the planar interface, and the device exhibits a photovoltaic effect. A PCE of 0.2% and external quantum efficiency (EQE) of 1.5% were estimated for this layered device. In another work, Lee and coauthors introduced graphene contacts for the monolayers MoS_2_ and WSe_2_ to improve the charge collection in the graphene-sandwiched 2-D heterojunction [43]. This monolayer heterojunction device exhibits a high EQE value of 2.4%. Also, Gong et al. synthesized a lateral WS_2_/MoS_2_ heterostructure showing an open-loop voltage and closed-loop current of 0.12 V and 5.7 pA, respectively [44]. Similarly, Duan et al. demonstrated a lateral WS_2_/WSe_2_ photodiode with external and internal quantum efficiencies of 9.9% and 43%, respectively [45].

Phosphorene, the monolayer of black phosphorus, is an intrinsic p-type semiconductor with high carrier mobility and a small bandgap suitable for broadband photodetectors [46, 47]. Phosphorene has been demonstrated to serve as the light absorber and charge transfer layer in solar cells, with enhanced light absorption and electron recombination. Deng et al. reported an electrically tunable black phosphorus monolayer MoS_2_ van der Waals heterojunction [48], which shows a maximum rectification ratio of ∼10 [5], a maximum responsivity of 418 mA/W, and an EQE of 0.3% with a back gate voltage of -30 V under 633 nm laser illumination. In the above-mentioned devices, substantial gain usually implies a slower device response. As a result, it is essential to optimize the gain and response time simultaneously for different applications. Furthermore, efforts are required to improve further the performance of 2-D monolayer material-based solar cells, including enhancement of light absorption, charge collection, and efficient exciton dissociation.

### 2.2. Thermoelectric Devices

Thermoelectric (TE) energy generation based on semiconductor materials has attracted immense attention, as the process can be directly used to convert waste heat into electricity. In general, the efficiency of a thermoelectric material to convert heat into electrical energy is characterized by the dimensionless figure of merit ZT = *σα*2*T*/*κ*, where *σ*, *α*, *κ*, and *T* are electrical conductivity, Seebeck coefficient, thermal conductivity, and absolute temperature, respectively [49]. Monolayer and few-layer 2-D materials present promising electrical and thermal properties as efficient TE materials [50–53].

Graphene has both high electric and thermal conductivities, a combination not ideal for thermoelectric devices. Compared with semimetal graphene, 2-D semiconductors are expected to enable better thermoelectric performance, since the highest thermoelectric power factor always resides in the degenerate semiconductor region. From first-principles DFT calculation, Huang et al. have investigated the thermoelectric performance of different dichalcogenide monolayers, including MoS_2_, MoSe_2_, WS_2_, and WSe_2_ [52]. They found that among these materials, p-type monolayer MoS_2_ has the highest first peak value of ZT at room temperature, with ZT > 0.5. As temperature increases, the first peak values of ZT increase linearly except for monolayer n-type WSe_2_/MoSe_2_ and p-type WS_2_. Naghavi et al. found that monolayer Pd_2_Se_3_ is a promising 2-D thermoelectric material with ultralow lattice thermal conductivity and high power factor [50]. A detailed analysis of third-order interatomic force constants reveals that the anharmonicity and soft phonon modes associated with covalently bonded [Se_2_]^2–^ dimers lead to ultralow lattice thermal conductivities in Pd_2_Se_3_ monolayers (1.5 and 2.9 W m^–1^ K^–1^ along the *a*- and *b*-axes at 300 K, respectively), comparable to those of high-performance bulk thermoelectric materials such as PbTe.

Buscema et al. found a large and tunable photothermoelectric effect in the monolayer MoS_2_ [53]. Unlike in many other semiconductors, the photocurrent generation in monolayer MoS_2_ is dominated by the photothermoelectric effect and not by the separation of photoexcited electron-hole pairs across the Schottky barriers at the MoS_2_/electrode interfaces. They also estimated the Seebeck coefficient for monolayer MoS_2_, finding a significant range between −4 × 10^2^ and −1 × 10^5^ *μ*V K^–1^, which can be tuned by an external electric field. This large and tunable Seebeck coefficient of monolayer MoS_2_ may lead to new applications such as on-chip thermoelectric devices and waste thermal energy harvesting. Wu et al. reported on electrical and thermopower measurements of monolayer CVD-grown MoS_2_ over a wide temperature range (20–300 K) by employing microfabricated heaters and thermometers (Figure 1(g)) [54]. Large values of up to ∼30 mV/K at room temperature were observed, which is two orders of magnitude larger than that in pristine graphene and also one order of magnitude larger than that of bulk MoS_2_. The thermopower is strongly dependent on temperature and applied gate voltage with a substantial enhancement at the vicinity of the conduction band edge. Pu et al. studied the thermoelectric properties of large-area CVD monolayers MoS_2_ and WSe_2_ and determined their thermoelectric properties in a FET configuration with a channel length of ~400 *μ*m [55]. With ionic gating in the electric double-layer transistor (EDLT) configuration, a large ∣*S*∣ (>200 *μ*VK^−1^) and power factor (>200 *μ*Wm^−1^ K^−2^) were observed in both monolayers MoS_2_ and WSe_2_. These results from monolayer TMDs show an order of magnitude enhancement in the power factor as a function of *σ* compared to their bulk counterparts, due to the quantum well-induced staircase-like density of states in 2-D TMDs. The notable enhancement represents a myriad of possibilities for monolayer TMD-based thermoelectric applications.

Recently, black phosphorus (BP) has attracted much attention for TE applications [56]. BP has been reported to retain a significant Seebeck coefficient (335 *μ*V K^−1^ at RT), a high carrier mobility (1000 cm^2^ V^−1^ s^−1^ at RT), and a layer-dependent bandgap, which can be tuned from 0.3 to 2 eV with decreasing thickness from bulk to monolayer. Saito et al. measured the gate-tuned Seebeck coefficient in 40 nm BP [57]. The Seebeck value reached 510 *μ*V K^−1^ in the hole-depleted state, which is much larger than the value for a bulk single crystal. The trend of the Seebeck value as a function of carrier concentration, however, was found to deviate from the first-principles calculation results. They attributed the large Seebeck value to the effective reduction of channel thickness in the depletion mode operation of the field effect transistor. This consideration suggests that a few- or monolayer BP single crystal might be capable of producing even larger thermoelectric power. In addition, other 2-D materials have been found to possess higher ZT than their bulky counterparts. Lee et al. found that 2-D SnS_2_ has higher electrical conductivity and lower thermal conductivity than its bulky counterpart (Figures 1(h) and 1(i)) [58]. The negative correlation between the enhanced electrical conductivity and reduced thermal conductivity as the thickness of SnS_2_ decreased is expected to be beneficial for thermoelectric nanogenerators. The Seebeck coefficient obtained for 2-D SnS_2_ nanosheets was 34.7 mV K^−1^ for 16 nm thick samples at 300 K. The layered structure materials including SnS_2_ can be cleaved down to few- or monolayer, with significant changes to the electrical and optical properties such as indirect-to-direct bandgap transition. A significant amount of efforts, both theoretically and experimentally, are required to realize the practical applications of 2-D material-based thermoelectric energy-harvesting technologies.

### 2.3. Piezoelectric Nanogenerators

Piezoelectricity widely exists in materials with polarization domains or noncentrosymmetric structures, allowing reversible conversion between electrical and mechanical signals. Harvesting mechanical energy by piezoelectric nanogenerators (PENGs) has received numerous attentions owing to the diverse flexibility of its sophisticated design to directly convert mechanical energy into electricity for many integrated applications. With the advancement of material synthesis at the molecular level, 2-D monolayer materials are of great interest in high-performance piezoelectric nanogenerators [59–61].

**Figure 2:**
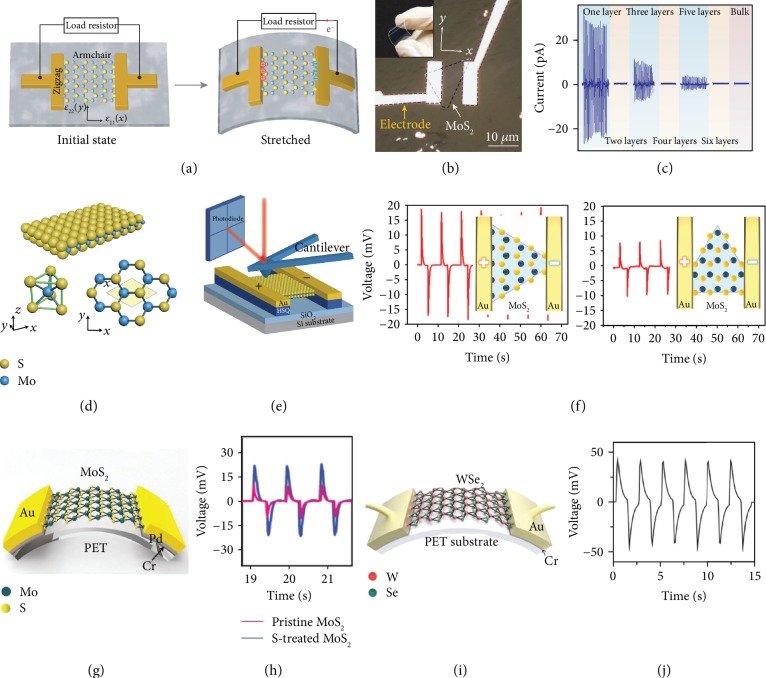
Piezoelectric nanogenerators based on monolayer 2-D materials. (a–c) Operation scheme of PENG based on the monolayer MoS_2_. The transparent device with monolayer MoS_2_ shows the highest output performance. Reproduced with permission from the Nature Publishing Group [62]. (d, e) Probing the piezoelectric property of free-standing monolayer MoS_2_. Reproduced with permission from the Nature Publishing Group [63]. (f) Voltage output performance determined by different directions of MoS_2_ grown by the CVD method on a Si substrate. Reproduced with permission from Elsevier Ltd. [64]. (g, h) Sulfur treatment enhanced the output performance of PENG based on the monolayer MoS_2_. Reproduced with permission from Wiley-VCH [65]. (i, j) PENG based on mono/bilayer WSe_2_ synthesized via CVD and turbostratic stacking. Reproduced with permission from Wiley-VCH [66].

Due to the strain-induced lattice distortion and the associated charge polarization, monolayer MoS_2_ is theoretically predicted to be strongly piezoelectric, an effect that disappears in the bulk counterparts because of their centrosymmetric structures. On this basis, Wu and coworkers have made the first experimental observation of piezoelectricity in a monolayer MoS_2_ flake and demonstrated a transparent and flexible PENG (Figures 2(a) and 2(b)) [62]. A monolayer MoS_2_ device under 0.53% strain can generate a voltage of 15 mV and a current of 20 pA, corresponding to a power density of 2 mW/m^2^ and a 5.08% mechanical-to-electrical energy conversion efficiency. Furthermore, the evolution of the output performance was investigated with an increased number of atomic layers (*n*) in MoS_2_ flakes. As shown in Figure 2(c), cyclic stretching and releasing of thin MoS_2_ flakes with an odd number of atomic layers produce oscillating piezoelectric voltage and current outputs, whereas no output is observed for flakes with an even number of layers. These results confirm that monolayer MoS_2_ with broken inversion symmetry has a strong intrinsic piezoelectric response, whereas centrosymmetric bilayers and bulk crystals are nonpiezoelectric. The existence of piezoelectricity in free-standing monolayer MoS_2_ has also been proved by atomic force microscopy measurement (Figures 2(d) and 2(e)) [63]. Results demonstrate that monolayer MoS_2_ exhibits a piezoelectric effect with a coefficient of 2.9 × 10^−10^ C/m, which is in good agreement with the theoretical value and comparable to widely piezoelectric wurtzite structure materials (e.g., ZnO).

Inspired by these pioneering works, the study of piezoelectricity in 2-D materials has attracted increasing interests. Kim et al. fabricated a PENG based on CVD-grown monolayer MoS_2_ and compared the output performance with different orientations (Figure 2(f)) [64]. The measurement results showed that the maximum outputs of 20 mV and 30 pA for the armchair direction and 10 mV and 20 pA for the zigzag direction were generated under 0.5 Hz and 0.48% strain, respectively. Han et al. demonstrated a sulfur (S) vacancy passivated monolayer MoS_2_ PENG (Figure 2(g)) [65]. Both pristine MoS_2_ monolayer and S-treated MoS_2_ monolayer were used in fabricating PENGs, and the performance of PENGs was compared before and after S-treatment. After S-treatment, the output peak current and voltage of the monolayer MoS_2_-based PENG were increased by more than three times (100 pA) and two times (22 mV), respectively (Figure 2(h)). Further, the S-treatment increases the maximum power by almost ten times. The significant enhancement in output performance is attributed to the reduction of the charge carrier density of the monolayer MoS_2_ surface by S-treatment. Thus, the screening effect of piezoelectric polarization charges by the free carrier is significantly reduced.

Besides MoS_2_, other monolayer 2-D TMD materials, such as MoSe_2_, MoTe_2_, WS_2_, WSe_2_, and WTe_2_, are also found to have piezoelectricity [59]. The magnitude of the piezoelectricity strongly depends on the number of layers, usually disappearing or significantly diminishing when more than two layers are present. Lee et al. have reported a simulation and experimental observation of the piezoelectricity in mono-/bilayer WSe_2_ synthesized via CVD and turbostratic stacking (Figures 2(i) and 2(j)) [66]. A monolayer WSe_2_-based PENG under 0.39% strain generates a voltage of 45 mV and a short-circuit current of 100 pA. The maximum instantaneous power reached 2.54 pW, and the device can sustain for over 1000 cycles. In general, the bilayer WSe_2_ with Bernal stacking loses its piezoelectricity due to the centrosymmetric structure in this stacking mode. The authors also fabricated the bilayer WSe_2_ via turbostratic stacking, which retains its piezoelectricity. The PENG exhibits excellent mechanical stability at a strain of up to 0.95% and generates an output voltage of 85 mV. The robust piezoelectricity of 2-D WSe_2_ makes it potential candidates for mechanical sensors, actuators, and energy harvesters for wearable electronics.

Theoretical exploration of the piezoelectricity in a broad range of 2-D materials has also achieved fruitful results. In addition to TMDs, other monolayer 2-D materials were found to have a piezoelectric effect based on the first-principles calculation. Fei et al. predicted an anisotropic piezoelectric effect in monolayer group IV monochalcogenides (SnSe, SnS, GeSe, and GeS) [67]. W. B. Li and J. Li found that several group III monochalcogenides, including GaS, GaSe, and InSe, are piezoelectric in their monolayer form [68]. Recently, an intensive data mining over 50,000 inorganic crystals was identified by Cheon et al., and 325 potential 2-D piezoelectric monolayers were found [69].

### 2.4. Triboelectric Nanogenerators

Triboelectrification can be used to convert mechanical energy into electricity in combination with electrostatic induction. In 2012, Fan et al. invented the first flexible triboelectric nanogenerator (TENG) [14], which presents a cost-effective and sustainable strategy to convert mechanical energy into electricity. Since then, the development of TENG is surprisingly fast with the invention of various new models and the application of new materials and novel designs. In principle, any materials, including 2-D materials, with distinct charge affinity can be used to construct an efficient TENG. Here, we only review the recent advances made in the TENGs based on monolayer 2-D materials, focusing on their structure, performance, and future applications.

**Figure 3:**
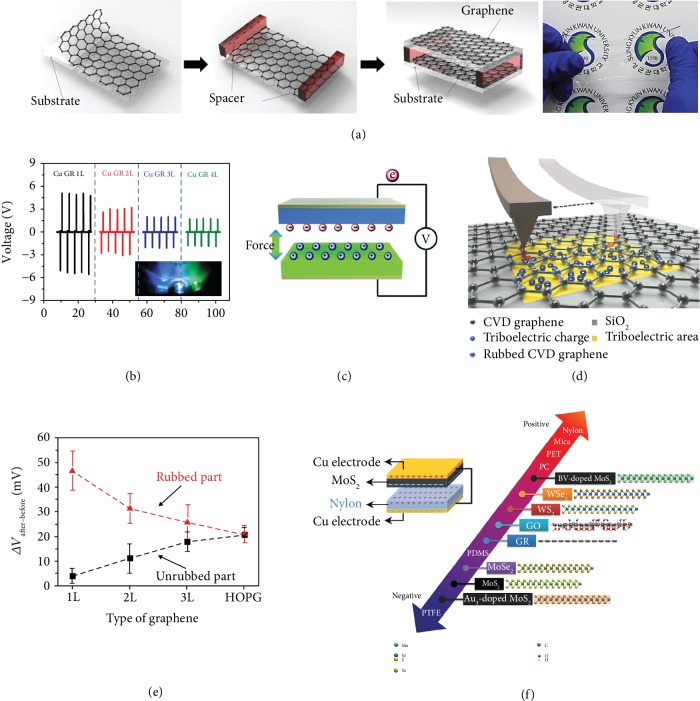
Triboelectric nanogenerators based on monolayer 2-D materials. (a, b) Design and device fabrication of transparent TENG used monolayer graphene as the electrode. The monolayer graphene-based device exhibited the highest output performance. Reproduced with permission from Wiley-VCH [26]. (c) Mechanism of power generation of a triboelectric nanogenerator. Reproduced with permission from the Royal Society of Chemistry [70]. (d, e) Tunnelling triboelectrification by friction of the monolayer graphene with a Pt AFM tip. The average surface potential decreases as the number of graphene layers increases. Reproduced with permission from the Nature Publishing Group [73]. (f) Triboelectric series of 2-D layered materials determined by the performance of TENGs. Reproduced with permission from Wiley-VCH [75].

Graphene not only can store the electric charge induced by triboelectrification but also can be used as the conductive electrode for the electric output in TENGs. Kim et al. reported a transparent flexible TENG using monolayer CVD-grown graphene as both the friction layer and output electrode [26]. The device consisted of two graphene-polyethylene terephthalate (PET) films separated by a plastic spacer, forming a gap of ~0.8 nm between the upper PET and lower graphene (Figure 3(a)). When the PET layer and the graphene layer come into contact under pressing or bending, triboelectric charges with opposite signs will be induced on their surfaces, and the electrons will transfer from the surface of graphene to the surface of PET. As the external force is released, the charged surfaces will be separated, and an electric potential difference will be built between the two graphene electrodes. The current will flow between the two graphene electrodes through the external load to screen out the electric field built up by the charged surfaces (Figure 3(c)) [70]. Such monolayer graphene flexible device exhibited a high output voltage and an output current density of 5.0 V and 500 nA cm^−2^, respectively. The output voltage and output current were found to decrease with an increasing number of graphene layers. Average output voltage values of 3.0, 2.0, and 1.2 V and average output current density values of 250, 160, and 100 nA cm^−2^ were observed for the 2-layer, 3-layer, and 4-layer graphene-based devices, respectively (Figure 3(b)). These results confirm that monolayer graphene is a promising candidate for high-performance TENG with excellent flexibility and stretchability for powering future low-power portable devices and electronics. Subsequently, Chandrashekar et al. reported a large-area method for preparing monolayer graphene-based TENGs through a roll-to-roll transfer of CVD-grown graphene to plastic substrates [71]. Such wearable transparent TENG shows an output voltage and current density of 22 V and 0.075 *μ*A cm^−2^, respectively.

The mechanism of contact electrification (CE) in TENGs has been investigated with a nanoscale spatial resolution using atomic force microscopy (AFM) and Kelvin probe force microscopy (KPFM). Very recently, Z. L. Wang and A. C. Wang systematically studied the fundamental mechanism of CE using KPFM for the solid-solid case and concluded that that electron transfer is the dominant mechanism for CE between two solid pairs [72]. For monolayer graphene, Kim et al. revealed that the triboelectric charging behavior is likely due to the tunneling triboelectrification phenomenon [73]. They rubbed monolayer graphene with a Pt-coated AFM tip and found that parts of the triboelectric charges tunnel through graphene and are trapped at the interface among the air-gap and the insulation layer, as shown in Figure 3(d). For monolayer graphene, tunneling triboelectrification prevails because of graphene's ultimate thinness and the extremely high speed of tunneling processes. By contrast, when increasing the number of layers, tunneling becomes more and more unlikely, and triboelectric charges tend to spread over the entire graphene layer and, consequently, increase or decrease the potential of the entire graphene layer (Figure 3(e)). This work has proved the function of the tunneling triboelectrification in a large-scale integration of independent 2-D field-effect transistor and also opens up a new way to fabricate rewritable 2-D electronics.

Recently, Zou et al. introduced a universal standard method to quantify the triboelectric series for a wide range of polymers [74]. Using the device and measurement of TENG, Seol et al. investigated the triboelectric charging behaviors of various 2-D layered materials, including MoS_2_, MoSe_2_, WS_2_, WSe_2_, graphene, and graphene oxide in a triboelectric series, with nylon as the counter material (Figure 3(f)) [75]. The results show that MoS_2_ exhibits the highest output voltage and current values reaching up to 7.48 V and 0.82 *μ*A, respectively, indicating that MoS_2_ is triboelectrically the most negative among the 2-D materials. Further research confirmed that the effective work functions of the 2-D materials are one of the main factors dictating their triboelectric charging behaviors. This study provides new insights to utilize 2-D monolayer materials in flexible TENG devices.

### 2.5. Hydrovoltaic Devices

**Figure 4:**
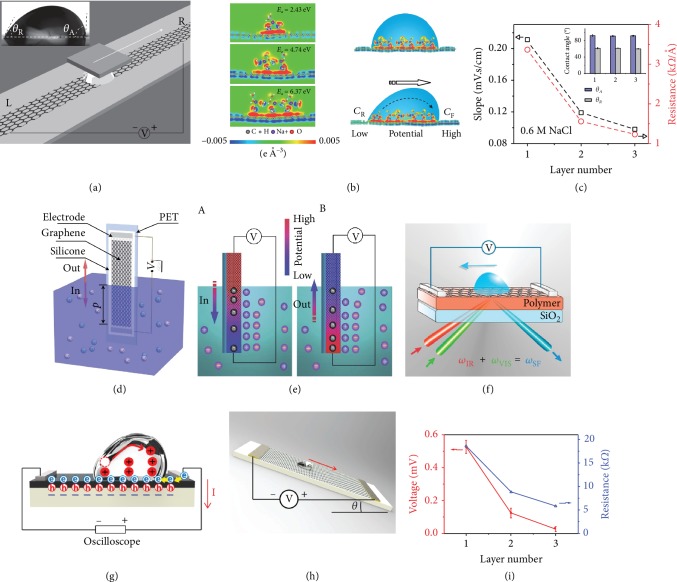
Hydrovoltaic devices for harvesting water kinetic energy on a monolayer graphene surface. (a) Generating electricity by moving a droplet of ionic liquid along the monolayer graphene and a SiO_2_/Si wafer. (b) DFT results for the distribution of charges at the graphene/solution interface and potential difference induced by moving a droplet. (c) Drawing potential and resistance change with graphene layers. Reproduced with permission from the Nature Publishing Group [79]. (d) Schematic illustration for waving potential by moving graphene across the surface of saltwater. (e) Schematic of the electric double-layer and its boundaries of ion adsorption (A) and desorption (B) on the surface of graphene during the inserting and pulling out processes. Reproduced with permission from the Nature Publishing Group [80]. (f) Sum-frequency vibrational spectroscopy for the mechanism study of electric power generation from ionic droplet motion on polymer-supported graphene. Reproduced with permission from the American Chemical Society [83]. (h) Triboelectrification-induced large electric power generation from a single moving droplet on graphene/PTFE. Reproduced with permission from the American Chemical Society [81]. (i) Measuring the electrical response to a water flow over graphene PVDF heterointerface. Output voltage and resistance change with number of graphene layers. Reproduced with permission from Wiley-VCH [82].

Water contains tremendous energy in a variety of forms, yet very little of this energy has been harnessed. Wang et al.'s group developed various approaches for harvesting water wavy energy using TENGs [76, 77]. On the other hand, nanostructured materials can generate electricity upon interaction with water through the hydrovoltaic effect [27, 78]. In 2014, Yin and coworkers demonstrated that a potential of a few millivolts could be produced by moving a droplet of ionic water over a monolayer graphene strip [79]. As shown in Figure 4(a), a voltage of ∼0.15 mV is produced by moving a droplet of 0.6 M NaCl solution on the surface of monolayer graphene at a constant velocity of 2.25 cm s^−1^. Density functional theory calculations show that hydrated Na^+^ cations are adsorbed on the graphene surface because of its high positive adsorption energy (over 2 eV), while hydrated Cl^−^ anions are repulsive to graphene due to its negative adsorption energy. With an increasing number of adsorbed hydrated Na^+^, a thin layer of accumulated electrons is distributed along the contact surface of graphene, forming a double-layer pseudocapacitor at the graphene-solution solid-liquid interface. For a static NaCl droplet on graphene, the charge symmetrically distributed on both sides of the droplet, and thereby no potential is produced. When moving the droplet on the graphene, Na^+^ ions are adsorbed at the front end, charging the pseudocapacitor forward and drawing electrons in the graphene (Figure 4(b)). Meanwhile, ions are being desorbed at the rear of the droplet, discharging the pseudocapacitor and releasing the electrons to the graphene. The moving process of the droplets causes an unbalanced distribution of internal ions, inducing a potential on the monolayer graphene. As the droplets slide back and forth over the surface of the monolayer graphene, the induced voltage drives the electrons to flow in the external circuit to output electrical energy. Interestingly, it is shown that the slopes of the voltage-velocity curves for the monolayer, bilayer, and trilayer graphene samples decrease in proportion to the square resistance of the samples (Figure 4(c)).

Compared with water droplets, waving water is more abundant and contains much more energy, which can be harvested through the generated wave potential. Yin et al. further found that the motion of monolayer graphene in/out the ionic solutions could generate electricity [80]. When monolayer graphene on the PET substrate is inserted into an ionic solution, a distinct voltage can be induced without the pressure gradient (Figure 4(d)). Pulling the graphene out of the water surface produces an inverse voltage. This waving potential is proportional to the inserting velocity and the sheet size. A 2 × 10 cm^2^ sized graphene sheet can generate an open-circuit voltage of up to 0.1 V with a short-circuit current of 11 *μ*A at a velocity of 1 m∙s^−1^. When inserting a bilayer or trilayer graphene sheet of the same size into the NaCl solution, the induced voltage becomes one order lower than that in monolayer graphene, mainly owing to a significant reduction in the square resistance of these multilayered graphene samples. The electric double-layer (EDL) theory was proposed to explain the energy-harvesting mechanism. Similar to the above-mentioned ion-adsorption process, the positive adsorption energy of Na^+^ ions and negative adsorption energy of Cl^−^ ions lead to Na^+^ ions adsorbing on the graphene surface, forming the EDL that consists of the firmly adsorbed Na^+^ ions and attracted Cl^−^ ions at the graphene-solution interface. During the insertion process, the moving boundary of the EDL draws more electrons from graphene than in the equilibrium, raising local hole concentration and electric potential (Figure 4(e), A). Once the graphene sheet is completely immersed, the moving boundary disappears, and the voltages in all the subsections drop abruptly to a baseline value. Similarly, pulling out the graphene sheet drives the EDL boundary to move downwards and reverses the hole current (Figure 4(e), B). The voltage and current can be scaled up through series or parallel connection of multiple graphene devices.

Subsequent experiments show that the waving and drawing potentials largely depend on the substrate underneath the graphene due to the strong charge interaction between monolayer graphene and the substrate. In other words, the nature of the substrate will critically influence the charge interaction between graphene and the liquid droplet for generating the electric power output. Kwak et al. demonstrated that the power output from moving a water droplet on monolayer graphene supported by polytetrafluoroethylene (PTFE) substrate could generate an output voltage, current, and power of ∼0.45 V, ∼4.8 *μ*A, and ∼1.9 *μ*W, respectively, which exhibits a 100-fold enhancement compared to previous studies [81]. Such enhancement was explained using the change in triboelectrification-induced pseudocapacitance between a water droplet and the monolayer graphene on PTFE. On the surface of the PTFE substrate, a strong negative potential is generated by the triboelectrification between PTFE and deionized water. The triboelectric potential lead to the accumulation of positive and negative charges on the top and bottom surfaces of graphene, respectively (Figure 4(g)). Besides, it should be noted that the output voltage for bilayer and trilayer graphene on PTFE is significantly reduced to around 1.2 mV, due to the screening of a substrate effect in the multilayer graphene.

Besides the triboelectrification, the piezoelectric effect of the substrate can also be used to enhance the performance of the hydrovoltaic devices. Lin and colleagues showed that moving a droplet on monolayer graphene supported by piezoelectric polyvinylidene fluoride (PVDF) substrate introduces an extra charge on the substrate surface due to the pressure of the droplet (Figure 4(h)) [82]. The introduction of the PVDF beneath graphene results in a voltage output up to 0.1 V even with deionized water due to the enhanced charging by the piezoelectric effect. Because the screening effect of the water for the piezoelectric charges becomes smaller with few-layer graphene, the flow-induced voltage decreases rapidly when bilayer or trilayer graphene was used (Figure 4(i)). Recently, Yang et al. used sum-frequency vibrational spectroscopy to study the interactions at the aqueous solution/graphene/polymer interface and found a different mechanism (Figure 4(f)) [83]. They proposed that the monolayer graphene does not attract ions and only plays the role of transporting carriers and ions in droplets. The surface dipole layer of the polymer substrate attracted the ions to move and generate electricity. A higher potential would be produced if a stronger surface dipole was established on the polymer substrate, such as PVDF. The exact mechanism underpinning the electricity generation by the hydrovoltaic devices requires further study to explore the water-material interaction and surface/substrate effects.

### 2.6. Other Emerging Techniques

**Figure 5:**
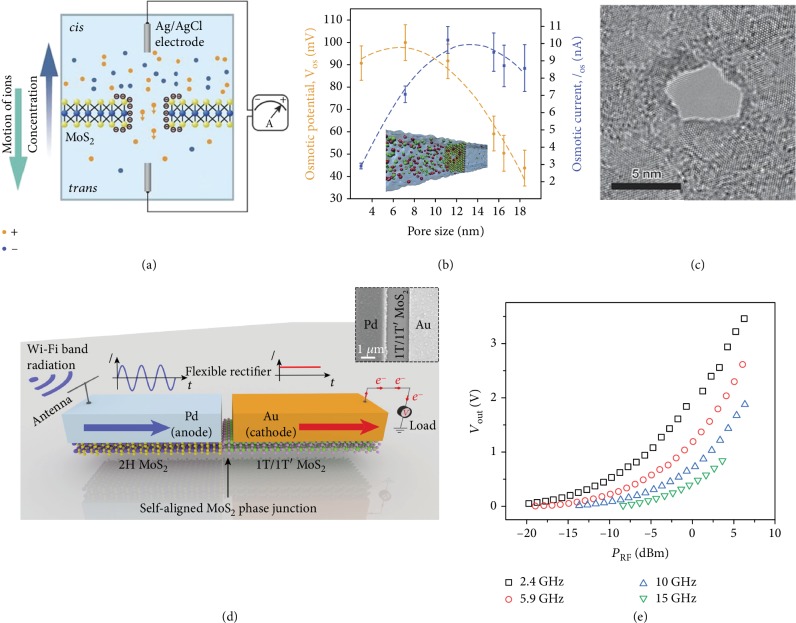
Monolayer MoS_2_-enabled devices for the osmotic energy and Wi-Fi wireless energy harvesting. (a) Mechanism of monolayer MoS_2_ nanopores as a nanopower generator. (b, c) Osmotic potential and current as a function of pore size and TEM-drilled MoS_2_ nanopore of a 5 nm diameter. Reproduced with permission from the Nature Publishing Group [87]. (d) Flexible rectenna based on a monolayer 2-D MoS_2_-heterostructure Schottky diode. (e) Output voltage as a function of the input RF power delivered to the device at four different frequencies. Reproduced with permission from the Nature Publishing Group [89].

Besides the hydrokinetic energy of ocean waves, another attractive and clean way to generate power from blue energy is to convert the energy released by the salinity gradient potential into electricity [84–86]. This form of energy is derived from the difference in osmotic pressure that is generated when a semipermeable membrane separates two aqueous solutions with different salt concentration. A chemical potential gradient arises at the interface of these two liquids and drives ions spontaneously across the membrane, forming an osmotic ion flux towards the equilibrium state. Feng et al. have developed an osmotic nanopower generator by utilizing monolayer MoS_2_ with nanopores as a semipermeable membrane (Figure 5(a)) [87]. The osmotic power is generated by separating two reservoir KCl solutions of different concentrations with a freestanding MoS_2_ membrane with the thickness of 0.65 nm and a typical nanopore diameter in the range of 2-25 nm. The highly negatively charged surface enables selective K^+^ ion flow, resulting in a net osmotic current. The device performance is related to the salt concentration and pH of the solution, as well as the pore size. As shown in Figures 5(b) and 5(c), small pores display better voltage behavior, reflecting improved performance regarding the ion selectivity. The power density of a single-layer MoS_2_ membrane with a uniform pore size of 10 nm and a porosity of 30% has been estimated to be as high as 10^6^ W m^–2^, which is six orders of magnitude higher than the power density obtained by reverse electrodialysis with classical exchange membranes made of cellulose acetate or polyamide.

However, these demonstrations of highly efficient power conversion have been performed in extremely alkaline conditions (pH 11) to increase the surface charge of the material and improve the ion selectivity. To overcome this drawback, the same researchers investigated the possibility of tuning the surface charge of monolayer MoS_2_ nanopores with light and demonstrated a light-induced efficiency boost for osmotic power harvesting [88]. By modulating the surface charge with light illumination, the ion selectivity of the membrane can be raised by a factor of 5 while staying at a neutral pH. Increased surface charge at the pore rim enhances the ion selectivity and therefore enables a larger osmotic voltage (dominating in small pores), while the increased surface charge of the overall membrane enhances the surface conductance and therefore the osmotic current (dominating in larger pores). This work demonstrates the potential of the MoS_2_ nanopore power generator to be applied in the natural environment. In the future, blue energy relies on the chemical potential difference generated between solutions and the hydrokinetic energy of ocean waves would provide a sustainable energy source for large-scope applications.

Nowadays, Wi-Fi is becoming increasingly ubiquitous in both indoor and outdoor environments, especially with the rise of the Internet of Things (IoT) applications. Electromagnetic radiation from Wi-Fi systems as an always-on radio frequency (RF) energy would be ideal for powering future distributed electronics. Very recently, Zhang et al. demonstrated an atomically thin and flexible ultrafast Schottky diode based on a monolayer MoS_2_ semiconducting-metallic-phase heterojunction, which can convert energy from Wi-Fi signals into electricity for powering electronics (Figure 5(d)) [89]. The rectenna uses a radio frequency antenna to capture electromagnetic waves carrying Wi-Fi in the form of alternating current (AC) waves. Then the AC Wi-Fi signal travels into the flexible 2-D MoS_2_ phase junction and is converted into a direct current (DC) voltage that could be used to power electronic circuits. Figure 5(e) shows the output voltage increases as the input RF power delivered to the device increases at four different frequencies. At an input RF power of about 5 mW, an output voltage of 3.5 V can be achieved. In the Wi-Fi band (2.4 GHz), the maximum output efficiency for the current device stands at 40 percent, compared to 50-60 percent for traditional rectennas made from more expensive and rigid silicon or gallium arsenide. This work may lead to the development of future high-speed flexible platform that can be used for wirelessly charging future ubiquitous electronics using the existing Wi-Fi infrastructure as an energy hotspot.

## 3. Monolayer Heterostructures and Hybrid Devices

The weak van der Waals (vdW) interlayer interaction allows us to isolate 2-D monolayers and restack them into arbitrary stacking heterojunctions without the need to consider the atomic commensurability and crystal lattice matching. This type of monolayer heterostructures, named van der Waals heterostructures (vdWHs), has been applied in many electronic and optoelectronic devices such as field-effect transistors (FETs), photodetectors, and sensors, and energy-harvesting devices such as solar cells and thermoelectric devices [90–92]. Regarding the applications of 2-D vdWHs for solar cells and thermoelectric conversion, we have listed some typical devices in the above section. Besides, it allows considerable freedom in integrating monolayer 2-D materials with various nanoscale materials to create heterostructures or hybrid devices with functions that were not previously possible [93–95]. In this section, we will highlight some recent advances in developing heterostructured devices with monolayer 2-D materials and other materials for energy harvesting.

**Figure 6:**
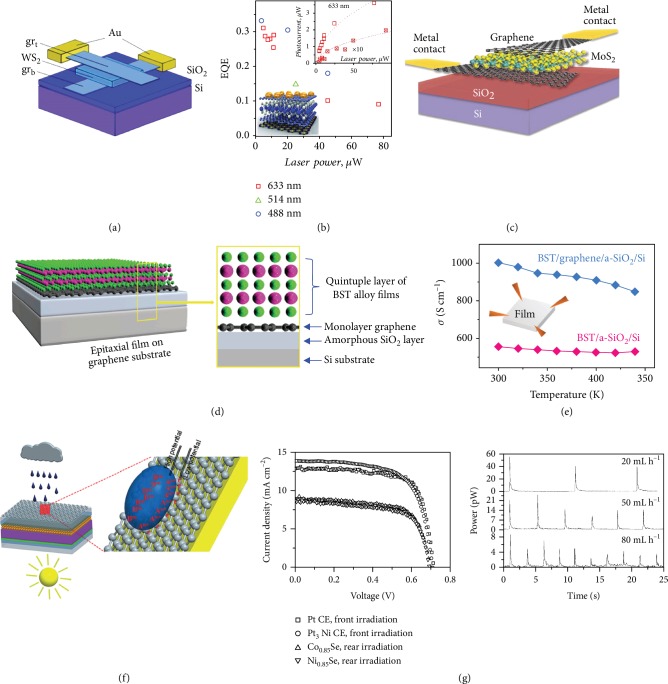
Heterostructures and hybrid devices for energy harvesting. (a, b) Design and performance of an efficient heterogeneous solar cell consisting of a thin layer of WS_2_ within two layers of graphene. Reproduced with permission from AAAS [96]. (c) Schematic of a heterostructure (graphene/MoS_2_/graphene) photovoltaic device. Reproduced with permission from AAAS [22]. (d, e) Epitaxial growth of the BST film on monolayer graphene shows a higher thermoelectric performance compared to an individual BST film. Reproduced with permission from Wiley-VCH [97]. (f, g) Structure and output performance of a hybrid nanogenerator that can produce electricity from rain and sun. Reproduced with permission from Wiley-VCH [98].

Numerous possibilities exist for creating heterostructures by stacking 2-D materials with differing properties. The TMDs, such as WS_2_, MoS_2_, and WSe_2_, display insulating, semiconducting (with band gaps in the visible region of the spectrum), and metallic behavior and can enable novel device architectures. Britnell et al. sandwiched a thin layer of WS_2_ within two layers of graphene to form an efficient Schottky diode-like solar cell (Figure 6(a)) [96]. In this device, the semiconducting WS_2_ functions as the active energy-harvesting material, whereas the metallic graphene works as a transparent electrode for the efficient collection of photogenerated carriers. An extrinsic quantum efficiency (EQE) above 30% and a photoresponsivity above 0.1 A/W were achieved in such TMD/graphene stacks. The EQE did not appear to be dependent on wavelength, as shown in Figure 6(b). The decrease in quantum efficiency with increasing power is due to screening of the built-in electric field by the excited electrons in the conduction band of WS_2_. To enhance the optical electric field, gold nanospheres were applied on top of heterostructures, which enhanced the optical field in the active layer and allowed for a 10-fold increase in the photocurrent. Similar structures can be extended to other 2-D materials, such as MoS_2_ and chemically functionalized graphene, which highlights the enormous potential of heterostructures in solar energy harvesting (Figure 6(c)) [22].

Kim et al. recently demonstrated a thermoelectric energy device using the epitaxial Bi_0.5_Sb_1.5_Te_3_ (BST) on monolayer graphene by pulsed laser deposition [97]. As shown in Figure 6(d), in the hybrid film, monolayer graphene promoted stable epitaxial growth of a BST film with better crystallinity, lower defect density, and higher thermoelectric performance than an individual BST film. The values of the specific conductivity in the plane (*σ* ≈ 1003 S cm^−1^) and the Hall mobility of charge carriers of the BST film at room temperature are both significantly higher than those of the same film on the SiO_2_/Si substrate without graphene (Figure 6(e)). The Seebeck coefficient value of the hybrid BST film is increased compared to those of pure BST film or single crystal and ingot. Graphene plays a crucial role in this hybrid thermoelectric generator and significantly improves the thermoelectric performance of the heterostructured device.

Recently, hybrid energy harvesters have also received considerable attention in the field of renewable and sustainable energy harvesting for their potential in effectively harvesting multiple types of environmental energy. Tang et al. demonstrated a hybrid device combining carbon nanomaterials with dye-sensitized solar cells to harvest energy from rain and sun (Figure 6(f)) [98]. The solar cells can be excited by sunlight on sunny days, and the graphene on the surface can harvest mechanical kinetic energy of raindrops on rainy days as a hydrovoltaic device. Apart from a front power conversion efficiency of 6.53% and a rear efficiency of 4.26% under simulated AM1.5 sunlight, the device can yield a current of 0.49 *μ*A and voltage of 109.26 *μ*V for each raindrop (Figure 6(g)). Similar functions can be achieved by hybrid devices consisting of triboelectric nanogenerators and solar cells [99] for persistently generating electricity in all weathers.

## 4. Conclusions and Perspectives

In this review, we have briefly reviewed the recent progress on energy-harvesting devices built with monolayer 2-D materials, with a focus on various unique devices for solar, thermal, and mechanical energy harvesting. The use of monolayer 2-D materials has led to significant advances in energy harvesting because the distinctive characteristics of these materials can not only improve the output performance but also drive many unique applications, such as the osmotic pressure and Wi-Fi wireless energy. Still, the advancement of related fields is challenged by the fact that the output performance of a single device is at a relatively low level, and the interaction is limited to surfaces of the monolayer. The wide range of 2-D materials available for exploration could usher in more new mechanisms and device structures. With substantial progress already being made, we anticipate significant advances in both the fundamental understanding and technological development of 2-D monolayer material-based energy-harvesting devices. To achieve this goal, we envision that the following research challenges need to be addressed, which are also accompanied by abundant research opportunities:

1. The scalable production of large-area and integrated devices: the first main challenge is related to the large-scale production of 2-D materials and devices with well-controlled properties, e.g., interfaces and surfaces. The ongoing efforts are limited by the vague potential in scaling up (e.g., low yield and random flake shape through exfoliation), stringent restrictions in growth substrates and conditions (e.g., high temperature, high vacuum, gas control in chemical vapor deposition, or molecular beam epitaxy), small sizes, and/or instability of synthesized materials. This challenge is expected to be solved through the holistic design of materials and cooptimization of the device and fabrication for enabling fundamentally new material technology that can produce scalable, substrate-agnostic, high-performance 2-D energy materials [100, 101]
2. Improving efficiency and output performance: the extensive understanding of the mechanism of energy harvesting and charge transfer will facilitate the optimization of the output performance of devices to achieve a higher energy conversion efficiency. Also, the development of new energy-harvesting methods based on new principles is attractive and critical
3. Improving stability and environmental adaptability: due to the atomic thickness of the active material and the wide distribution of the device in the environment, 2-D material devices need to adapt to a variety of environmental conditions and maintain stable performance. The optimization of material properties, device structure, and the incorporation of packaging process would address this challenge
4. Integrating the energy harvesters for practical, killer applications: the increasing demand for practical applications is the main driving force for the exploration of new energy-harvesting processes. A future study should be steered to develop devices with different functions to meet various application needs, such as implantable soft bioelectronics and wearable electronics. The flat, ultrathin nature of 2-D materials also allows for potential integration in the vertical direction, leading to a 3-D architecture with diverse functionalities and boosted performance compared to planar counterparts. Still, issues such as the electrical interconnection across layers and the reliability/robustness of the related manufacturing process need to be properly addressed [102, 103]
5. The confluence of many emerging and “traditional” disciplines, e.g., nanomanufacturing, data science, material science, solid-state electronics, and chemistry, is expected to lead to more theoretical and experimental advances in exploring 2-D energy materials, as well as usher in abundant research and development opportunities for implementing novel 2-D energy devices and systems
